# Analytical performance of a multi-target open real-time PCR assay for simultaneous detection of tuberculosis, non-tuberculous mycobacteria, and drug resistance in a high-burden setting

**DOI:** 10.1128/spectrum.01643-26

**Published:** 2026-08-15

**Authors:** Zeeshan Sidiq, Ankita Anand, Payal Tyagi, Kaushal Kumar Dwivedi, Sanjay Rajpal, Kamal Kishore Chopra

**Affiliations:** 1 New Delhi Tuberculosis Centre 534397, New Delhi, Delhi, India; ICON plc, London, United Kingdom

**Keywords:** tuberculosis, non-tuberculous mycobacteria, multiplex real-time PCR, rifampicin resistance, isoniazid resistance, molecular diagnostics

## Abstract

Timely diagnosis of tuberculosis (TB) and drug resistance is crucial for effective disease control. Multiplex open molecular platforms capable of simultaneously detecting *Mycobacterium tuberculosis* complex (MTBc), non-tuberculous mycobacteria (NTM), and resistance to first-line anti-TB drugs could significantly streamline diagnostic pathways. We evaluated two multiplex real-time PCR assays (MTBc/NTM R-Gene and MTB-RIF/INH R-Gene) using 300 well-characterized samples (150 MTBc-positive and 150 MTBc-negative). Composite reference standards, including culture, MPT64 antigen testing, and line probe assays and phenotypic drug susceptibility testing. The MTBc detection demonstrated 100% sensitivity and specificity (150/150). NTM detection showed 70.0% sensitivity (35/50) and 100% specificity, indicating limitations in NTM species coverage. Rifampicin resistance was detected with 96.0% sensitivity (48/50) and 100% specificity. Isoniazid resistance detection achieved 100% sensitivity and specificity (50/50). High agreement (κ = 0.76–1.00) with established reference standards was observed. This analytical validation confirms that multiplex open real-time PCR assays accurately and simultaneously detect MTBc, NTM, and rifampicin and isoniazid resistance using culture isolates. Clinical validation is warranted. While these platforms offer potential advantages in flexibility and expanded resistance profiling, further studies on clinical diagnostic accuracy, cost-effectiveness, and operational feasibility are essential to determine their practical utility and programmatic impact in high-burden settings.

**IMPORTANCE:**

Despite major advances in molecular diagnostics, critical gaps persist in tuberculosis (TB) detection, particularly the limited identification of isoniazid resistance and inadequate differentiation from non-tuberculous mycobacteria. In this study, a multiplex open real-time PCR assay demonstrated high analytical accuracy for simultaneous detection of *Mycobacterium tuberculosis* complex, rifampicin and isoniazid resistance, with strong agreement to reference standards. By integrating multi-target detection within a single workflow, this approach addresses key limitations of current diagnostic strategies that rely on rifampicin as a surrogate for multidrug resistance. The open-platform design further offers flexibility, scalability, and reduced dependence on proprietary systems, which are critical considerations in high-burden settings. These findings highlight the potential of next-generation molecular diagnostics to improve early resistance detection, refine treatment decisions, and strengthen TB control efforts globally.

## INTRODUCTION

Tuberculosis (TB) remains a global public health challenge, with India bearing the highest burden of disease. (1) Rapid, accurate, and scalable diagnostic tools are becoming increasingly important in meeting the TB elimination targets set by the National Tuberculosis Elimination Programme (NTEP). Traditional diagnostic approaches are limited: smear microscopy lacks sensitivity, and culture, while regarded as the gold standard, is slow and resource-intensive (2–4).

The nucleic acid amplification tests have substantially improved TB diagnostics, allowing for rapid detection of *Mycobacterium tuberculosis* and rifampicin resistance. Cartridge-based molecular platforms have been widely adopted due to their operational simplicity and faster turnaround time (5–8). However, these systems are mostly proprietary, have high per-test costs, and primarily target rifampicin resistance as a surrogate marker for multidrug-resistant TB (MDR-TB). Their limited ability to detect isoniazid resistance independently may result in underdiagnosis of isoniazid mono-resistance and poor treatment recommendations. Furthermore, issues like indeterminate results, reliance on continuous supply chains, and restricted flexibility in assay design continue to hamper their effectiveness in routine programmatic settings (9, 10).

Distinguishing *Mycobacterium tuberculosis* complex (MTBc) from non-tuberculous mycobacteria (NTM) remains a key diagnostic challenge, with NTM becoming more common in clinical practice (11, 12). Misclassification can lead to incorrect treatment and poor patient outcomes. The most widely used rapid molecular assays do not significantly address this issue, highlighting the need for more comprehensive diagnostic approaches. (13) Importantly, currently available rapid molecular platforms do not provide a single-step solution for simultaneous MTBc-NTM differentiation along with detection of both rifampicin and isoniazid resistance, highlighting a critical unmet diagnostic need.

In this context, open real-time polymerase chain reaction (real-time PCR) platforms represent a novel approach that enables simultaneous multi-target detection within a single workflow. These systems enable not only differentiation of *Mycobacterium tuberculosis* complex (MTBc) from NTM, but also the identification of mutations associated with drug resistance, including both rifampicin and isoniazid. By utilizing non-proprietary reagents and customizable assay designs, open-platform real-time PCR systems provide advantages in terms of cost, adaptability, and throughput. Importantly, this multi-target capability represents a significant advancement over most existing cartridge-based platforms, which are typically limited to MTBc detection and rifampicin resistance alone.

Despite these advantages, there are limited clinical data on the performance of open real-time PCR-based assays for the simultaneous detection of TB, NTM, and drug resistance in real-world settings (14, 15). Most existing studies have focused on closed, cartridge- or chip-based platforms, leaving the applicability of multi-target open systems underexplored. In contrast, open-platform assays allow for the simultaneous detection of MTBc, differentiation of NTM, and identification of both rifampicin and isoniazid resistance within a single open-platform workflow. This integrated capability directly addresses two major limitations of current diagnostic algorithms: underdiagnosis of isoniazid mono-resistance and inadequate identification of NTM in routine clinical practice.

To our knowledge, there is limited independent evaluation of such multiplex open-platform assays combining species identification and dual drug resistance detection. In this context, the present study was undertaken to evaluate the analytical accuracy of the Argene MTBc/NTM and MTB-RIF/INH qualitative real-time PCR assays (bioMérieux, India) for the detection of the *Mycobacterium tuberculosis* complex, differentiation from non-tuberculous mycobacteria, and identification of rifampicin and isoniazid resistance using well-characterized culture isolates. The analytical performance of these assays was assessed using culture and a line probe assay as reference standards, with the aim of generating evidence of their potential role as comprehensive molecular diagnostic tools in high-burden settings.

## MATERIALS AND METHODS

### Study design, population, and reference standard

This laboratory-based, comparative analytical validation study was conducted at the New Delhi Tuberculosis Centre, New Delhi, an Intermediate Reference Laboratory under the NTEP, India. The study aimed to assess the analytical performance of open real-time PCR assays for the detection of MTBc, differentiation from NTM, and detection of drug resistance.

A total of 300 well-characterized culture isolates were included, comprising microbiologically confirmed laboratory isolates. All isolates were maintained in liquid culture using the BACTEC MGIT 960 system. Samples were stratified into MTBc-positive (*n* = 150) and MTBc-negative (*n* = 150) groups. Although this distribution does not reflect clinical prevalence and may affect predictive values, this 1:1 ratio was deliberately chosen to ensure robust calculations of analytical sensitivity and specificity for each target, a common practice in initial analytical validation studies. The MTBc-positive group consisted of culture-confirmed *Mycobacterium tuberculosis* isolates. Identification of MTBc from positive liquid cultures was confirmed using MPT64 antigen testing. These isolates had predefined drug susceptibility profiles based on GenoType MTBDRplus VER 2.0 Line Probe Assay (Hain Lifescience GmbH, Nehren, Germany), which targets the rpoB gene for rifampicin resistance and katG and inhA genes for isoniazid resistance. These comprised two independent drug-specific subsets: 50 rifampicin-resistant isolates and 50 isoniazid mono-resistant isolates, along with 50 drug-susceptible isolates. The rifampicin-resistant subset comprised 49 multidrug-resistant and 1 rifampicin mono-resistant isolate, whereas the isoniazid-resistant subset consisted entirely of mono-resistant isolates. Among the isoniazid-resistant isolates, katG mutations were identified in 39 (78%) cases, while inhA promoter mutations were detected in 11 (22%). For drug resistance performance evaluation, two independent subsets of MTBc isolates were analyzed for each drug, each comprising 50 resistant and 50 susceptible isolates. Corresponding phenotypic drug susceptibility testing results obtained using the BACTEC MGIT 960 system were available for these isolates as part of prior routine laboratory characterization. The MTBc-negative group comprised 50 NTM culture isolates and 100 samples negative for mycobacteria, confirmed by culture and/or molecular methods such as Xpert MTB/RIF.

NTM isolates were identified using the GenoType Mycobacterium CM/AS line probe assay (Hain Lifescience GmbH, Nehren, Germany) for mycobacterial speciation. The species distribution included *Mycobacterium abscessus* (*n* = 6), *Mycobacterium chelonae* (*n* = 4), *Mycobacterium mageritense* (*n* = 2), *Mycobacterium fortuitum* (*n* = 7), *Mycobacterium kansasii* (*n* = 10), *Mycobacterium szulgai* (*n* = 8), *Mycobacterium intracellulare* (*n* = 6), *Mycobacterium scrofulaceum* (*n* = 4), *Mycobacterium avium* (*n* = 2), and *Mycobacterium avium complex* (*n* = 1).

Drug resistance classification for rifampicin (RIF) and isoniazid (INH) was performed independently for each drug based on previously established genotypic results obtained using the GenoType MTBDRplus VER 2.0 line probe assay, which served as the primary reference standard. A composite reference framework incorporating antigen-based and molecular methods was applied for classification. Phenotypic drug susceptibility testing data were used as supportive evidence to enhance the robustness of diagnostic classification.

The sample size was selected to enable reliable estimation of diagnostic accuracy parameters. The distribution of samples across resistance profiles and non-tuberculous mycobacteria was structured to ensure adequate representation for subgroup analyses and meaningful performance evaluation.

#### Index tests and procedures

Two multiplex real-time PCR assays (ARGENE, bioMérieux, India) were evaluated: (i) MTBc/NTM R-Gene for species identification and (ii) MTB-RIF/INH R-Gene for detection of resistance-associated mutations. Both assays utilize 5′-exonuclease (TaqMan) chemistry.

For sample processing, 500 µL of liquid culture from previously characterized positive MGIT tubes (BACTEC MGIT 960 system) was centrifuged at 5,000 rpm for 15 minutes, and the pellet was resuspended in 200 µL of phosphate-buffered saline (pH 8.0). An internal control (IC2) was added prior to extraction to monitor extraction efficiency and PCR inhibition. DNA extraction was performed using the QIAamp DNA Mini Kit (QIAGEN, Hilden, Germany; Cat. No.: 51304) from 200 µL of processed sample, following the manufacturer’s instructions. DNA was eluted in 50 µL of Buffer AE and used immediately or stored at −20°C until further analysis. As the assay is validated for use with positive MGIT cultures, no prior standardization of bacterial load was performed.

#### Real-time PCR amplification

PCR reactions were prepared in a dedicated clean area. Each reaction consisted of 15 µL of assay-specific master mix and 10 µL of extracted DNA. Positive and negative controls were included in each run. The positive control comprised an *MTBc* IS6110 fragment cloned into a plasmid vector, while the negative control consisted of nuclease-free water processed alongside samples to monitor contamination, handling errors, and PCR inhibition (amplifying only the internal control target and not MTB-specific targets). Amplification was performed on a Bio-Rad CFX96 real-time PCR system using the following cycling conditions: initial denaturation at 95°C for 15 minutes, followed by 42 cycles of 95°C for 10 seconds and 60°C for 40 seconds, with fluorescence acquisition at each elongation/hybridization cycle.

The MTBc/NTM R-Gene assay targets IS6110 and IS1081 for MTBc detection and the rpoB gene for pan-mycobacterial detection. The MTB-RIF/INH R-Gene assay detects mutations within the rifampicin resistance-determining region of *rpoB* (codons 511, 516, 522, 526, and 531), and mutations in *katG* (including codon 315 and 328) and the *inhA* promoter region associated with isoniazid resistance. The evaluated assays are designed to detect a defined subset of clinically relevant NTM species; species falling outside this predefined panel are not expected to be identified. Fluorescence detection was performed across multiple channels in accordance with the manufacturer’s instructions, including FAM (MTBc-specific targets, rifampicin and INH high-level resistance targets), HEX (internal control), ROX (pan-mycobacterial), and the Cy5 channel (INH low-level resistance targets).

Assay performance characteristics, as defined by the manufacturer, include limits of detection of approximately 15 CFU/mL for MTBc and 0.1 CFU/mL for NTM, and 5 copies/µL for resistance targets. Cycle threshold (Ct) cut-offs of ≤37 cycles for MTBc/NTM detection and ≤42 cycles for resistance detection were applied.

#### Interpretation and analysis

Results were interpreted according to manufacturer-defined criteria. MTBc was defined by detection of MTBc-specific targets, whereas NTM was defined by detection of pan-mycobacterial targets in the absence of MTBc-specific amplification. Resistance to RIF and INH was inferred from detection of mutation-specific signals. Each run was considered valid only if control reactions met predefined criteria. Cycle threshold values were generated using instrument software CFX Maestro Version 2.3. Index test results were compared against the composite reference standard, comprising MPT64 antigen testing for MTBc identification, line probe assays for NTM speciation and initial resistance characterization, and phenotypic drug susceptibility testing (MGIT 960) as confirmatory evidence for resistance.

## RESULTS

During the study period, 300 culture-confirmed samples were analyzed, including 150 MTBC-positive and 150 MTBc-negative samples. The MTBC-negative group included 50 NTM isolates and 100 samples with no mycobacteria. Among the MTBc-positive isolates, 50 were rifampicin-resistant, 50 were isoniazid-resistant, and 50 were drug-susceptible, as determined by the GenoType MTBDRplus VER 2.0 LPA. For drug resistance analysis, a drug-wise approach was adopted using two independent subsets of MTBc isolates, each comprising 50 resistant and 50 susceptible isolates with valid results for the respective drug. Among the rifampicin-resistant isolates (*n* = 50), 49 (98%) were multidrug-resistant (MDR-TB), while 1 (2%) was rifampicin mono-resistant. In contrast, all isoniazid-resistant isolates (*n* = 50) were mono-resistant.

For MTBc detection, the assay identified all 150 MTBc-positive samples, corresponding to a sensitivity and specificity of 100%. Among the 150 MTBc-negative samples, there were no false-positive results. For NTM detection, the analytical sensitivity was lower at 70%, with 15 isolates not detected, whereas the specificity remained 100%. Of the 15 undetected isolates, 12 (80%) comprised *Mycobacterium abscessus* (*n* = 6), *Mycobacterium chelonae* (*n* = 4), and *Mycobacterium mageritense* (*n* = 2), which fall outside the assay’s defined detection scope; in addition, one isolate each of *Mycobacterium fortuitum*, *Mycobacterium kansasii*, and *Mycobacterium szulgai* was not detected despite being within the target range. The limited diversity of NTM species included in this analytical validation may impact the generalizability of this sensitivity. When restricted to NTM species within the assay’s defined detection range, the effective sensitivity was substantially higher. The positive predictive value was 100%, and the negative predictive value was 87%, indicating reliable identification when NTM was present, but some cases could be missed (Table 1).

For drug resistance detection, the assay correctly identified all 50 isoniazid-resistant isolates, with 100% sensitivity and specificity. Among the isoniazid mono-resistant isolates, katG mutations were observed in 39 (78%) cases, while inhA mutations were detected in 11 (22%). Rifampicin resistance was detected in 48 of 50 resistant isolates, giving 96% sensitivity, 100% specificity, and an overall accuracy of 98%. The positive and negative predictive values were high for both resistance targets, supporting confidence in resistance profiling (Table 1).

**TABLE 1:** Diagnostic accuracy of the Argene multiplex real-time PCR assay for MTBc/NTM identification and detection of rifampicin and isoniazid resistance^*a*^^,*b*^

Target	TP/TN/FP/FN	Sensitivity % (95% CI)	Specificity % (95% CI)	PPV % (95% CI)	NPV % (95% CI)	Accuracy % (95% CI)	Kappa
MTBc detection	150/150/0/0	100 (97.6–100)	100 (97.6–100)	100 (97.6–100)	100 (97.6–100)	100 (98.7–100)	1.00
NTM detection	35/100/0/15	70.0 (56.2–80.9)	100 (96.3–100)	100 (90.1–100)	87.0 (79.6–91.9)	90.0 (84.2–93.8)	0.76
Rifampicin resistance	48/50/0/2	96.0 (86.5–98.9)	100 (92.9–100)	100 (92.6–100)	96.2 (87.0–98.9)	98.0 (93.0–99.4)	0.96
Isoniazid resistance	50/50/0/0	100 (92.9–100)	100 (92.9–100)	100 (92.9–100)	100 (92.9–100)	100 (93.0–100)	1.00

^*a*^ All performance metrics are derived from analytical validation using culture isolates. The high accuracy and kappa values reflect strong analytical agreement within a controlled laboratory setting and may not directly translate to clinical performance on patient specimens due to spectrum bias.

^*b*^ MTBc, Mycobacterium Tuberculosis Complex; NTM, Non-Tuberculous Mycobacteria; True positives; TN, True negatives; FP, False positives; FN, False negatives; PPV, Positive predictive value; NPV, Negative predictive value.

**Fig 1:**
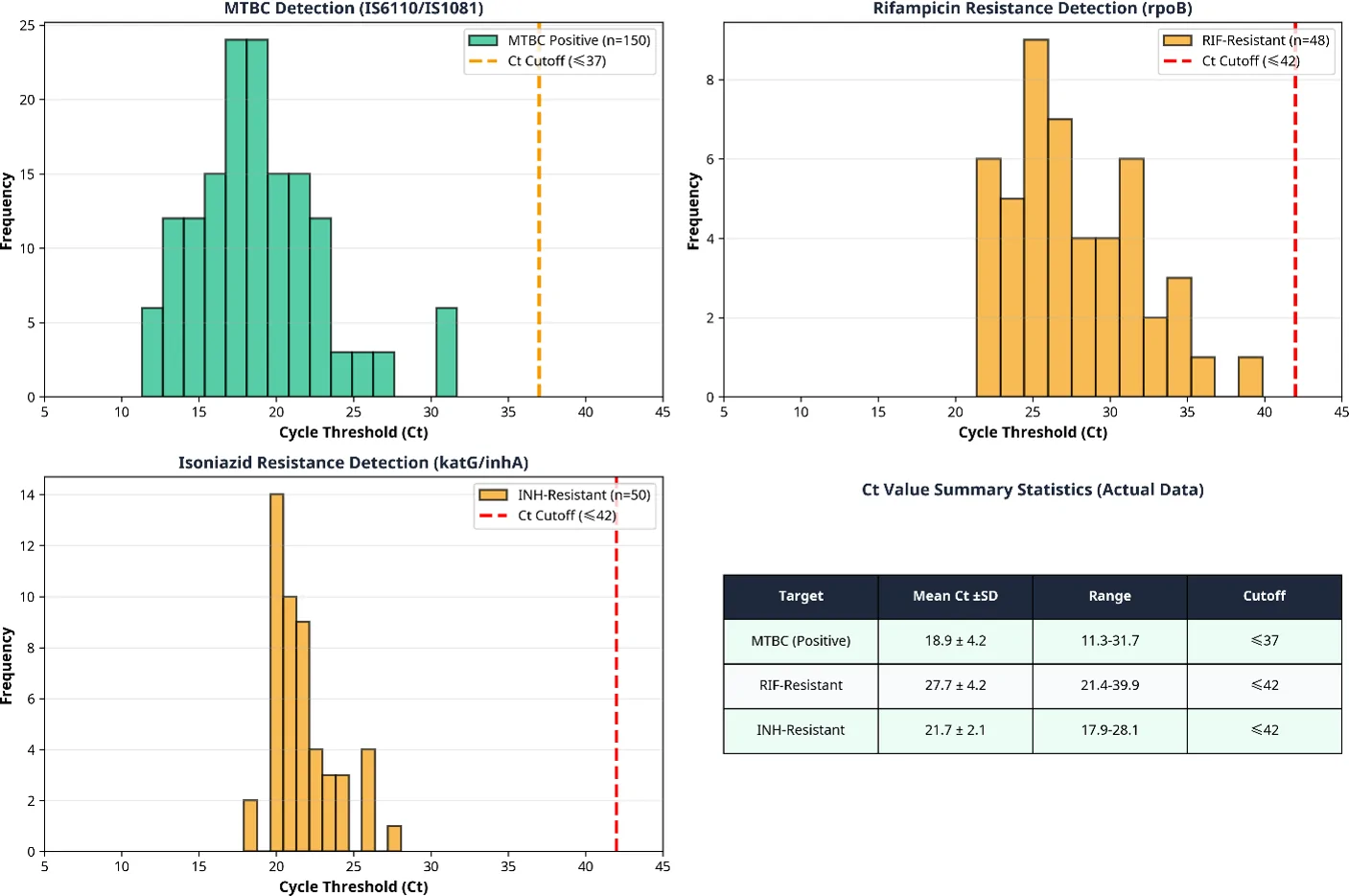
Distribution of Ct values for MTBc detection (IS6110/IS1081), rifampicin resistance (rpoB), isoniazid resistance (katG/inhA) targets, and summary statistics of Ct values. Histograms show the frequency distribution of Ct values for each target group, with each bar representing the number of isolates within a given Ct range. Lower Ct values indicate higher target DNA concentration, whereas higher Ct values reflect lower template abundance. Vertical dashed lines indicate the predefined assay Ct cut-offs for positivity (≤37 for MTBc and ≤42 for resistance targets). The accompanying table summarizes mean Ct values, standard deviations, and observed ranges for each target. Their limited ability to detect isoniazid resistance independently may result in underdiagnosis.

Ct value distributions across targets showed distinct ranges relative to assay cut-offs. MTBc targets demonstrated lower Ct values (mean 18.9; range 11.3–31.7) compared with resistance-associated targets. Rifampicin resistance (rpoB) showed higher Ct values (mean 27.7; range 21.4–39.9) within the assay detection threshold (≤42), while isoniazid targets (katG/inhA) showed a narrower distribution (mean 21.7; range 17.9–28.1). Ct values across all targets remained below their respective assay cut-offs, with no clustering near threshold levels (Fig. 1).

## DISCUSSION

This study demonstrates that a multiplex, open real-time PCR platform can achieve high analytical accuracy for the simultaneous detection of MTBc and key drug resistance markers, while also enabling differentiation from NTM within a single workflow using culture isolates. The high degree of concordance (κ = 0.76–1.00), indicating significant to near-perfect analytical agreement with established reference standards, further supports the analytical reliability of the assay in a controlled laboratory setting. The use of an enriched sample set with predefined resistance profiles, however, may limit the generalizability of predictive values and performance estimates in routine clinical settings.

The 100% sensitivity and specificity of the assay for MTBc detection underscore the reliability of nucleic acid amplification tests targeting conserved genomic regions. This performance is comparable to that of widely used molecular platforms, such as Xpert MTB/RIF, which have transformed early TB diagnosis and treatment initiation. In high-burden settings, where diagnostic delays fuel transmission and worsen patient outcomes, such rapid and accurate detection is critical (1, 5, 6). The absence of false-positive MTBc results is particularly important, as it minimizes the risk of inappropriate treatment and associated program burden. Ct value distributions were consistent across targets, with values remaining below assay cut-offs and no clustering near threshold levels, consistent with stable amplification performance.

The assay demonstrated outstanding agreement with reference standards for drug resistance detection. With just two false-negative results, rifampicin resistance was detected with 96% sensitivity and 100% specificity. This observation is consistent with the recognized limitations of molecular assays targeting specific rpoB regions, which may miss uncommon or atypical mutations outside of designated hotspots (16, 17). Nevertheless, this performance remains clinically robust given the role of rifampicin as a surrogate marker for multidrug-resistant tuberculosis. Most notably, the complete detection of isoniazid resistance (100% sensitivity and specificity), likely due to targeting both katG and inhA, represents a significant advance. In our study, INH-resistant isolates included mutations in both major target regions (39 *katG* and 11 *inhA*), indicating that the assay captured the predominant resistance mechanisms represented in the sample set. This capability addresses a critical gap in rifampicin-focused assays, which often underdiagnose isoniazid mono-resistance, potentially leading to suboptimal treatment regimens (9). By simultaneously detecting both key first-line drug resistances, this assay enables more precise treatment guidance and prevents the development of MDR-TB.

A key strength of this approach lies in its integrated, multi-target design on an open platform, enabling the concurrent detection of MTBc, differentiation of NTM, and identification of resistance-associated mutations. This is in contrast to the rapid molecular diagnostics currently in use, many of which may enhance diagnostic precision and may enhance diagnostic precision either depend on proprietary cartridge-based systems or are restricted to single-target detection. Open-platform assays offer potential advantages, including greater flexibility in assay design, reduced reliance on proprietary supply chains, and the possibility for cost optimization through reagent selection and workflow consolidation. These attributes are particularly relevant in high-burden settings, where expanding diagnostic capacity under national programs calls for scalable, adaptable, and sustainable solutions (10, 14, 18). The ability to customize assay panels may offer additional flexibility for resource-limited settings seeking to strengthen diagnostic infrastructure.

The observed 70% analytical sensitivity for NTM detection warrants careful interpretation. While high specificity (100%) ensures reliable positive results, some NTM infections may be missed. This limitation is inherent to the assay’s design, as the manufacturer specifies that it does not detect all known NTM species. The observed non-detection of certain NTM isolates should be interpreted in the context of assay design, as the majority belonged to species outside the defined detection scope, while the small number of missed target species likely reflects inherent limitations of molecular assays rather than a systematic performance deficiency. Such limitations are typical of multiplex assays with predefined detection panels reflecting the wide diversity of NTM and the lack of universally conserved molecular targets (5, 11). In this validation study, the diversity of NTM species was limited and may not represent the wider range encountered in routine clinical settings. In clinical practice, negative NTM results should be interpreted alongside clinical and microbiological findings, with additional methods such as culture or line probe assays remaining crucial in suspected cases. In clinical terms, missed NTM detection may delay appropriate species-specific treatment, reinforcing the need for complementary diagnostic tests. These findings highlight the broader diagnostic challenge of comprehensive NTM detection and underscore the need for continued refinement of molecular panels to expand species coverage (19, 20).

### Limitations

The limitations of this study include its reliance on well-characterized culture isolates rather than direct clinical specimens. While this approach is standard for initial analytical validation, it likely inflates estimates of diagnostic accuracy. Consequently, the reported performance represents an upper-bound analytical accuracy rather than true real-world clinical effectiveness. This distinction is critical for interpreting these findings and planning subsequent clinical validation studies. The impact of spectrum bias is particularly important for specific patient populations. For smear-negative and extrapulmonary tuberculosis, where a lower bacillary burden and the presence of inhibitors in clinical specimen can significantly reduce the sensitivity of molecular assays, the performance of this assay remains uncertian. We do not know how well this assay will perform in these critical patient groups and require dedicated clinical validation studies. Similarly, the impact of specimen type (e.g., sputum, cerebrospinal fluid, tissue, etc.), specimen quality, and storage conditions on assay performance has not been evaluated. Also, the use of a balanced sample distribution (1:1) may have influenced agreement statistics, so the κ values should therefore be interpreted in the context of study design. Furthermore, the limited diversity of NTM species may not fully reflect real-world epidemiology. As with all molecular assays, detection is confined to predefined genetic targets, potentially missing rare or novel resistance-conferring mutations. The distribution of INH resistance-associated mutations was limited to the major *katG* and *inhA* targets, and the assay’s performance against less common mutations could not be assessed. Finally, operational factors like how well this assay works with different types of specimens, how much time it takes to do hands-on work, indeterminate rates, and cost-effectiveness were not directly evaluated and need further study under programmatic settings. The performance of the assay in mixed infections (e.g., co-presence of MTBc and NTM) was not evaluated and warrants further investigation in clinical settings.

### Pathway to clinical validation

This analytical validation provides a necessary foundation for clinical validation studies. The next crucial step is to evaluate assay performance on direct clinical specimens from patients with suspected TB, including diverse specimen types and patient populations. Following successful clinical validation, implementation studies in reference laboratories and high-throughput diagnostic centers will be essential to assess programmatic impact, including effect on diagnostic turnaround time, treatment initiation, and patient outcome.

### Conclusion

This analytical validation demonstrates that multiplex open real-time PCR assays can achieve high analytical accuracy for simultaneous detection of MTBc, NTM differentiation, and identification of first-line drug resistance. The detection of isoniazid resistance addresses an important gap in widely used rapid molecular diagnostics. However, the translation of these analytical findings to clinical practice requires systematic clinical validation on direct specimens, assessment of performance in diverse patient populations, and evaluation of programmatic feasibility and cost-effectiveness. When strategically deployed following clinical validation, these platforms may enhance diagnostic precision and support more informed treatment decisions in high-burden settings.

## Data Availability

The data sets generated and/or analyzed during this study are available from the corresponding author on reasonable request.
